# Adherence to Mediterranean diet and dietary changes according to the fear of COVID-19 during the pandemic: a cross-sectional study

**DOI:** 10.1017/jns.2023.40

**Published:** 2023-05-08

**Authors:** Damla Gumus, Gulsum Gizem Topal, Sumeyra Sevim, Mevlude Kizil

**Affiliations:** Department of Nutrition and Dietetics, Hacettepe University Faculty of Health Sciences, Sıhhiye, Ankara 06100, Turkey

**Keywords:** COVID-19, Diet, Fear, Food, Mediterranean diet, BMI, body mass index, COVID-19, coronavirus disease 2019, FCV-19S, fear of COVID-19 scale, MD, Mediterranean diet, MEDAS, Mediterranean diet adherence scale, SARS-CoV, severe acute respiratory syndrome coronavirus, sd, standard deviation

## Abstract

The coronavirus disease 2019 (COVID-19) has led to the implementation of restrictions to reduce transmission worldwide. The restrictions and measures have affected the psychological health and eating habits. The objective of the present study was to evaluate dietary habits, lifestyle changes, adherence to the Mediterranean diet (MD) and fear of COVID-19 in Turkey during the pandemic. A cross-sectional online survey of socio-demographic characteristics, anthropometric measurements, nutrition, physical activity and lifestyle habits was used for data collection. The fear of COVID-19 levels of the participants was determined by the fear of COVID-19 scale (FCV-19S). The Mediterranean Diet Adherence Screener (MEDAS) was used to evaluate participants’ adherence to the MD. The differences between the FCV-19S and MEDAS according to gender were compared. Eight hundred and twenty subjects (76⋅6 % women and 28⋅4 % men) were evaluated within the study. The mean of MEDAS (ranged between 0 and 12) was 6⋅4 ± 2⋅1, and almost half of the participants moderately adhered to the MD. The mean of FCV-19S (ranged between 7 and 33) was 16⋅8 ± 5⋅7, while women's FCV-19S and MEDAS were significantly higher than men's (*P* < 0⋅001). The consumption of sweetened cereals, grains, pasta, homemade bread and pastries of the respondents with high FCV-19S were higher than in those with low FCV-19S. High FCV-19S was also characterized by decreased take-away food and fast food consumption in approximately 40 % of the respondents (*P* < 0⋅01). Similarly, women's fast food and take-away food consumption decreased more than men's (*P* < 0⋅05). In conclusion, the respondents’ food consumption and eating habits varied according to the fear of COVID-19.

## Introduction

The severe acute respiratory syndrome coronavirus (SARS-CoV) spread worldwide quickly and affected millions of people worldwide^(1)^. The measures and quarantines implemented to control the spread of ‘coronavirus disease 2019’ (COVID-19) pandemic caused major effects around the world^(2)^. Moreover, increased infection and mortality rates with other evolving variants of the coronavirus led to mental health problems such as anxiety, depression and stress^(3)^. One of the main stressors in the pandemic was the fear of being infected with the virus, defined as the fear of COVID-19^(4)^. However, it was not only the fear of death that triggers the fear of COVID-19, but also the anxiety caused by the uncertainty^(5)^.

The relationship between pandemic and nutrition is significant from different perspectives. Studies showed that nutrition and the immune system are essential in the progression of the disease^(6,7)^. Diet-related diseases such as obesity, cardiovascular diseases, diabetes and hypertension are also associated with the poor prognosis of COVID-19, and the importance of eating habits is emphasised^(8)^. In addition to the relationship between chronic diseases and nutrition, the effects of the pandemic on mood, mental health and emotional well-being can also change eating habits. Notably, impaired eating behaviour is more common in people with depression, anxiety and mood disorders^(9)^. Studies conducted during the pandemic revealed various and diverse changes in people's eating habits such as a decrease in nutritional quality of diet compared with the pre-pandemic period and an increase in the consumption of comfort foods or the transition to adopt a healthier diet^(10–12)^.

Recently, the relationship between the Mediterranean diet (MD) components and health has been among the most researched subjects^(13)^. Fruits, vegetables, legumes and olive oil, the main components of MD, are also rich sources of bioactive polyphenols. Polyphenols, especially flavonoids and their metabolites, have a protective effect on cardiovascular and metabolic diseases with their antioxidant, anti-inflammatory and antithrombotic properties^(14)^. Considering the inflammatory and antithrombotic effects associated with COVID-19, the MD becomes more critical with these characteristics. Since MD has numerous effects on health, a diet with high adherence to MD during the pandemic might be beneficial. On the other hand, fear and stress experienced during the pandemic might negatively affect healthy food consumption and the adaptation of healthy diets such as MD^(15–17)^.

The increased time people spend at home during the pandemic might cause mental, emotional and lifestyle problems. The secondary harms of the pandemic appear as malnutrition, social isolation, irregular sleep and a sedentary lifestyle. Considering the relationship between nutrition and COVID-19, it is crucial to evaluate the impact of the pandemic and the stress-causing disease in order to develop preventive and therapeutic nutritional strategies. Although there has been an interest in studying the changes in the eating habits during the pandemic, a limited number of studies have investigated the adherence to MD. In addition, studies examining the relationship between pandemic-induced fear and the adherence to are limited. Therefore, the present study aimed to examine the fear of COVID-19, adherence to the MD and dietary changes of Turkish individuals during the third wave of the pandemic.

## Methods

### Study design and participants

The study was conducted via an online survey with the participation of people aged 18–65 in Turkey during the third wave of the pandemic, where nationwide full lockdowns were implemented for reducing the spread of the virus. Researchers created the questionnaire via Google Form which could be answered by smartphone or computer based on the previous studies of Di Renzo *et al.*^(16)^ and Gornicka *et al.*^(18)^. The survey link was sent to the participants using convenience and snowball sampling via social media (Facebook, Instagram, Linkedin, Twitter and Whatsapp). The exclusion criteria were pregnancy or lactation. The study was conducted under the Declaration of Helsinki, and ethical approval was obtained from Hacettepe University Ethics Committee (Approval number: E-68552689-302.14-00001575445). Informed consent was obtained from all participants.

Overall, 860 participants were reached during the study period. Forty participants were excluded because of missing data such as weight, height, a lack of answers to scales and inconsistency with inclusion criteria. Hence, a total of 820 subjects were evaluated within the study.

### Data collection and the questionnaire

The online questionnaire consisted of socio-demographic characteristics, the fear of COVID-19 scale (FCV-19S), Mediterranean Diet Adherence Screener (MEDAS), self-reported anthropometric measurements (height and body weight), eating habits, physical activity and lifestyle habits. Individuals were asked to declare their consumption of certain foods, food groups and lifestyle habits comparing before and during the pandemic. Specific questions about dietary habits were modified from the survey conducted by Gornicka *et al.*^(18)^ which included foods such as vegetables, fruits, nuts, pasta and grains, homemade food/meals, wholegrain foods, bread, pastry, bakery products, sweets, cake, ice cream and puddings, sweetened cereals and/or cereal bars, processed meats, milk, dairy products, eggs, fish, marine products, legumes, white and red meat, fast foods, coffee, tea, water, sugary/sparkling beverages, wine/beer, other alcoholic beverages, snacks, honey, pekmez, garlic and spices/spicy sauces^(18)^. Respondents were also asked to declare the changes in the total food consumption and the difficulties in food availability. Respondents’ answers were re-categorised as follows: increased consumption (‘I eat more’); decreased consumption (‘I eat less’) and no changes (answers: ‘I eat the same’ or ‘I did not eat before and during the pandemic’) for the analysis of the data. In addition, the food mentioned above was categorised by separating into two groups based on the scoring of the MEDAS components^(19)^.

### Assessment of COVID-19 fear

The fear of COVID-19 levels of the participants was determined by using the FCV-19S developed by Ahorsu *et al.*^(20)^. The scale was adapted to Turkish by Bakioglu *et al.*^(21)^. It is a unidimensional scale with seven items which has a 5-point Likert-type rating (ranging from 1: Strongly disagree to 5: Strongly agree). Higher overall scores of the FCV-19S indicate a more severe fear of COVID-19. Cronbach's alpha internal consistency coefficient of the scale was found as 0⋅855 in the study.

Moreover, to assess the participants’ eating habits, lifestyle changes and food consumption according to the fear, the scores of FCV-19S were classified based on the mean ±1 standard deviation (sd) of FCV-19S. Accordingly, participants were classified into three groups: low (<Mean-sd), moderate (between Mean − sd and Mean + sd), and high (**>**Mean + sd) fear. The mean of FCV-19S was 16⋅8 ± 5⋅7; therefore, total scores were classified as follows: <11⋅1 was low, 11⋅1–22⋅5 was moderate and >22⋅5 was high levels of fear.

### Mediterranean diet adherence

The adherence to the MD was assessed using the MEDAS, adapted in Turkish^(22)^. The validated 14-item Mediterranean Diet Adherence Screener (MEDAS) ranges from 0 to 14 points^(19)^. Based on the MEDAS levels, participants were divided into three categories: (1) low adherence (scores between 0 and 5), (2) medium adherence (scores between 6 and 9) and (3) high (scores ≥ 10) adherence to the MD.

### Statistical analyses

All statistical analyses were performed using SPSS 25 statistical software (IBM Corp., Armonk, NY, USA). Descriptive statistics were calculated for all study participants. The Chi-square test was employed to assess the changes in eating and lifestyle habits, food and beverage consumption during the pandemic according to FCV-19S. *T*-test was used to compare the continuous variables between women and men, such as age, number of supplements used, BMI, the mean of FCV-19 and the mean of MEDAS. The mean of MEDAS according to FCV-19S during the pandemic was assessed using one-way ANOVA. The Spearman correlation coefficient was calculated to evaluate the correlation between FCV-19S and MEDAS.

## Results

General and social demographic characteristics of the population are shown in Table 1. Most of the respondents were women, and approximately 85 % lived together with their family members. 32⋅9 % of the respondents were employed in the same form as before the pandemic. One-fifth of the total respondents had at least one chronic disease. Approximately 20 % of the respondents stated that they got COVID-19 disease. Notably, 75 % of women declared using supplements, while 49 % of men declared taking supplements daily during pandemic (*P* < 0⋅001). Nearly 80 % of the respondents reported having no difficulty with food availability during the pandemic. Almost half of all the participants had normal body mass index (BMI). Furthermore, most of the women were in the normal BMI category, while most of the men were overweight. In addition, almost half of the participants moderately adhered to the MD. Moreover, the mean of FCV-19S was 16⋅8 ± 5⋅7, and women's FCV-19S and MEDAS were significantly higher than men's (*P* < 0⋅001).

**Table 1.** Participants’ general characteristics

	Total (*n* 820)	Female (*n* 587)	Male (*n* 233)	*P*-value
Age	31⋅3 ± 10⋅3	29⋅6 ± 8⋅8	35⋅6 ± 12⋅3	**<0⋅001**
Family composition				
Living alone	8⋅7 (71)	8⋅2 (48)	9⋅9 (23)	**<0⋅001**
Living partner	5⋅1 (42)	4⋅8 (28)	6⋅0 (14)	
Living partner and/or children	41⋅3 (339)	36⋅6 (215)	53⋅2 (124)	
Living with parents or other relatives	43⋅8 (359)	48⋅9 (287)	30⋅9 (72)	
Living with pet	1⋅1 (9)	1⋅5 (9)	0	
Employment forms during pandemic				
Did not work/unemployed	20⋅4 (167)	26⋅6 (156)	4⋅7 (11)	**<0⋅001**
Student	21⋅1 (173)	23⋅5 (138)	15⋅0 (35)	
Retired	3⋅4 (28)	1⋅0 (6)	9⋅4 (22)	
Began remote work and/or study	17⋅2 (141)	17⋅2 (101)	17⋅2 (40)	
Work in the same form as earlier	32⋅9 (270)	27⋅3 (160)	47⋅2 (110)	
Flexible/alternate working hours	1⋅8 (15)	1⋅0 (6)	3⋅9 (9)	
Reduction of working hours	3⋅2 (26)	3⋅4 (20)	2⋅6 (6)	
Have chronic disease	21⋅2 (174)	20⋅8 (122)	22⋅3 (52)	0⋅637
Use of supplements	68⋅3 (560)	75⋅6 (444)	49⋅4 (116)	**<0⋅001**
Number of supplements used	2⋅4 ± 2⋅8	2⋅7 ± 2⋅8	1⋅8 ± 2⋅8	**<0⋅001**
Got COVID-19	19⋅5 (160)	19⋅6 (115)	19⋅3 (45)	1⋅000
Difficulties with food availability				
No	77⋅6 (636)	75⋅8 (445)	82⋅0 (191)	0⋅063
Yes	22⋅4 (184)	24⋅2 (142)	18⋅0 (42)	
BMI	25⋅1 ± 14⋅6	24⋅6 ± 17⋅1	26⋅5 ± 3⋅7	**0⋅012**
Underweight	7⋅0 (57)	9⋅2 (54)	1⋅3 (3)	**<0⋅001**
Normal	53⋅0 (435)	61⋅2 (359)	32⋅6 (76)	
Overweight	27⋅9 (229)	20⋅3 (119)	47⋅2 (110)	
Obese	12⋅1 (99)	9⋅4 (55)	18⋅9 (44)	
The mean of FCV-19S	16⋅8 ± 5⋅7	17⋅4 ± 5⋅6	15⋅2 ± 5⋅5	**<0⋅001**
The mean of MEDAS adherence to MD	6⋅4 ± 2⋅1	6⋅6 ± 2⋅0	5⋅8 ± 2⋅0	**<0⋅001**
Low	32⋅0 (262)	28⋅8 (169)	39⋅9 (93)	**<0⋅001**
Moderate	52⋅4 (430)	52⋅8 (310)	51⋅5 (120)	
High	15⋅6 (128)	18⋅4 (108)	8⋅6 (20)	

Numerical data are presented as mean ±sd, categorical data as the number of persons (%).

According to the FCV-19S scores, 18 % of respondents were in low, 66 % of respondents were in moderate and 16 % of respondents were in a high level of fear groups. The MEDAS of respondents with low FCV-19S (5⋅9 ± 2⋅1) was significantly lower than the respondents with high and moderate FCV-19S (6⋅5 ± 2⋅0 and 6⋅5 ± 2⋅1, respectively) (*P* < 0⋅05). However, no significant correlation was found between FCV-19S and MEDAS (*r* = 0⋅061; *P* = 0⋅083).

Regarding the lifestyle changes during the COVID-19, nearly half of the respondents declared unchanged lifestyle (Table 2). However, approximately 40 % of the respondents with high FCV-19S claimed a worsened lifestyle (*P* < 0⋅001). Likewise, nearly half of the respondents with high FCV-19S stated that their total food consumption and appetite increased during the pandemic (*P* < 0⋅01). In addition, while approximately 40 % of respondents with high FCV-19S declared a change in the number of meals consumed per day, more than half of the respondents with low FCV-19S stated no change in the number of meals (*P* < 0⋅001). More than half of the respondents declared using supplements (Table 1), and notably, 33 % of respondents reported starting using supplements due to the pandemic. The most used supplements were vitamin D and C. Moreover, three-fourths of the total respondents declared increased screen time (Table 2).

**Table 2.** Changes in eating and lifestyle habits according to FCV-19S during the pandemic

	Level of FCV-19S				
	Total (*n* 820)	Low fear (*n* 148)	Moderate fear (*n* 541)	High fear (*n* 131)	*P*-value
The mean of MEDAS	6⋅4 ± 2⋅1	5⋅9 ± 2⋅1	6⋅5 ± 2⋅1	6⋅5 ± 2⋅0	**0⋅015**
Eating and lifestyle habits					
Became better	24⋅0 (197)	22⋅3 (33)	23⋅7 (128)	27⋅5 (36)	**<0⋅001**
No change	48⋅3 (396)	60⋅8 (90)	48⋅4 (262)	33⋅6 (44)	
Became worse	27⋅7 (227)	16⋅9 (25)	27⋅9 (151)	38⋅9 (51	
Total food consumption					
Ate less	12⋅1 (99)	9⋅5 (14)	12⋅2 (66)	14⋅5 (19)	**0⋅001**
No change	43⋅0 (368)	56⋅8 (84)	45⋅1 (244)	30⋅5 (40)	
Ate more	43⋅0 (353)	33⋅8 (50)	42⋅7 (231)	55⋅0 (72)	
Number of daily meals					
No changes	54⋅8 (449)	69⋅6 (103)	54⋅2 (293)	40⋅5 (53)	**<0⋅001**
Skipped one or more meals	10⋅2 (84)	5⋅4 (8)	10⋅4 (56)	15⋅3 (20)	**0⋅025**
Added one or more meals	10⋅4 (85)	8⋅8 (13)	9⋅6 (52)	15⋅3 (20)	0⋅128
Skipped snacks	6⋅6 (54)	4⋅7 (7)	5⋅9 (32)	11⋅5 (15)	**0⋅044**
Added one or more snacks	21⋅3 (175)	12⋅8 (19)	22⋅9 (124)	24⋅4 (32)	**0⋅019**
Appetite					
Decreased	14⋅5 (119)	12⋅8 (19)	14⋅8 (80)	15⋅3 (20)	**0⋅007**
No change	44⋅8 (367)	57⋅4 (85)	43⋅3 (234)	36⋅6 (48)	
Increased	40⋅7 (334)	29⋅7 (44)	42⋅0 (227)	48⋅1 (63)	
Weight					
No changes	31⋅8 (261)	38⋅5 (57)	31⋅1 (1689	27⋅5 (36)	0⋅340
Lost weight	18⋅2 (149)	16⋅9 (25)	17⋅6 (95)	22⋅1 (29)	
Gained a little weight	19⋅9 (163)	25⋅0 (37)	32⋅0 (173)	28⋅2 (37)	
Gained a lot of weight	30⋅1 (247)	19⋅6 (29)	19⋅4 (105)	22⋅1 (29)	
Supplement usage					
No change	66⋅8 (548)	69⋅6 (103)	359 (66⋅4)	65⋅6 (86)	0⋅724
Started to use	33⋅2 (272)	30⋅4 (45)	33⋅6 (182)	34⋅4 (45)	
Screen time					
Decreased	3⋅4 (28)	4⋅7 (7)	2⋅6 (14)	5⋅3 (7)	**<0⋅001**
No change	21⋅0 (172)	39⋅9 (59)	18⋅7 (101)	9⋅2 (12)	
Increased	75⋅6 (620)	55⋅4 (82)	78⋅7 (426)	85⋅5 (112)	

Numerical data are presented as mean ± sd, categorical data as the number of persons (%).

The bold indicates statistically significant values (*P* < 0⋅05).

Tables 3 and 4 represent the changes in food consumption according to FCV-19S classification. The results showed that during the pandemic, approximately 30 % of the respondents with both low and high FCV-19S increased their consumption of sweets (*P* < 0⋅05). The respondents with high FCV-19S were characterised by increased consumption of sweetened cereals or cereal bars in approximately 20 % of respondents; pasta, grains and homemade bread in about 30 % of respondents; homemade pastries in about 40 % of respondents (*P* < 0⋅05). Moreover, the food consumption mentioned above in the respondents with high FCV-19S was higher than those with low FCV-19S. This pattern with high FCV-19S was also characterised by decreased take-away food and fast food consumption in approximately 40 % of respondents (*P* < 0⋅01).

**Table 3.** Changes in positive MEDAS scored-foods consumption according to FCV-19S during the pandemic

	Level of FCV-19S			
Foods which have positive MEDAS score^†^	Low fear (*n* 148)	Moderate fear (*n* 541)	High fear (*n* 131)	*P*-value
Fresh fruits				
Decreased	8⋅8 (13)	10⋅7 (58)	15⋅3 (20)	0⋅474
Increased	33⋅1 (49)	30⋅1 (163)	29⋅8 (39)	
Dry fruits				
Decreased	9⋅5 (14)	13⋅5 (73)	14⋅5 (19)	0⋅227
Increased	23⋅6 (35)	18⋅7 (101)	25⋅2 (33)	
Fresh vegetables				
Decreased	3⋅4 (5)	8⋅5 (46)	7⋅6 (10)	0⋅139
Increased	30⋅4 (45)	33⋅8 (183)	37⋅4 (49)	
Frozen vegetables				
Decreased	14⋅2 (21)	14⋅4 (78)	12⋅2 (16)	0⋅952
Increased	8⋅1 (12)	9⋅4 (51)	9⋅2 (12)	
Nuts				
Decreased	4⋅7 (7)	8⋅7 (47)	11⋅5 (15)	0⋅053
Increased	38⋅5 (57)	33⋅6 (182)	42⋅7 (56)	
Pasta and grains				
Decreased	18⋅2 (27)	16⋅1 (87)	13⋅7 (18)	**0⋅001**
Increased	14⋅2 (21)	19⋅4 (105)	34⋅4 (20⋅9)	
Whole grain food				
Decreased	6⋅8 (10)	8⋅3 (45)	14⋅5 (19)	0⋅147
Increased	21⋅6 (32)	24⋅6 (133)	22⋅1 (29)	
Cow's milk and yogurt				
Decreased	6⋅8 (10)	7⋅0 (38)	7⋅6 (10)	0⋅946
Increased	31⋅8 (47)	34⋅2 (185)	35⋅9 (47)	
Cheese				
Decreased	6⋅8 (10)	6⋅1 (33)	3⋅8 (5)	0⋅510
Increased	26⋅4 (39)	32⋅0 (173)	34⋅4 (45)	
Other dairy products				
Decreased	6⋅1 (9)	7⋅2 (39)	8⋅4 (11)	0⋅483
Increased	21⋅6 (32)	25⋅3 (137)	29⋅8 (39)	
Eggs				
Decreased	6⋅8 (10)	7⋅9 (43)	11⋅5 (15)	0⋅611
Increased	29⋅7 (44)	29⋅8 (161)	31⋅3 (41)	
Fish				
Decreased	12⋅8 (19)	14⋅0 (76)	16⋅8 (22)	0⋅462
Increased	12⋅8 (19)	16⋅6 (90)	19⋅1 (259	
Marine products				
Decreased	12⋅8 (19)	11⋅5 (62)	14⋅5 (19)	0⋅831
Increased	6⋅8 (10)	7⋅9 (43)	6⋅1 (8)	
Legumes				
Decreased	7⋅4 (11)	9⋅1 (49)	9⋅9 (139	0⋅217
Increased	19⋅6 (29)	20⋅9 (113)	29⋅0 (38)	
White Meat				
Decreased	7⋅4 (11)	9⋅1 (49)	6⋅9 (9)	0⋅086
Increased	18⋅2 (27)	25⋅5 (138)	32⋅1 (42)	
Increased				
Garlic				
Decreased	6⋅1 (9)	5⋅7 (31)	6⋅9 (9)	0⋅619
Increased	25⋅7 (38)	32⋅2 (174)	29⋅0 (38)	
Spices/spicy sauces				
Decreased	6⋅1 (9)	8⋅5 (46)	8⋅4 (11)	0⋅383
Increased	21⋅6 (32)	27⋅0 (146)	29⋅8 (39)	

Data are presented as the number of persons (%).

† The foods which were given one score when their consumption increased based on the MEDAS scoring.

The bold indicates statistically significant values (*P* < 0⋅05).

**Table 4.** Changes in other food consumption according to FCV-19S during the pandemic

	Level of FCV-19S			
Foods which have negative MEDAS score^††^	Low fear (*n* 148)	Moderate fear (*n* 541)	High fear (*n* 131)	*P*-value
Red meat				
Decreased	8⋅8 (13)	9⋅8 (53)	7⋅6 (10)	0⋅630
Increased	21⋅6 (32)	22⋅6 (122)	28⋅2 (37)	
Processed meat				
Decreased	18⋅9 (28)	18⋅7 (101)	17⋅6 (23)	0⋅086
Increased	8⋅1 (12)	13⋅9 (75)	19⋅8 (26)	
Bread				
Decreased	14⋅9 (22)	22⋅7 (123)	19⋅8 (26)	0⋅070
Increased	14⋅2 (21)	14⋅6 (79)	21⋅4 (28)	
Homemade bread				
Decreased	10⋅8 (16)	11⋅1 (60)	10⋅7 (14)	**0⋅048**
Increased	16⋅2 (24)	22⋅0 (119)	31⋅3 (41)	
Homemade food				
Decreased	7⋅4 (11)	5⋅2 (28)	4⋅6 (6)	0⋅201
Increased	42⋅6 (63)	49⋅0 (265)	56⋅5 (74)	
Take-away food				
Decreased	21⋅6 (32)	37⋅5 (203)	40⋅5 (53)	**0⋅002**
Increased	18⋅2 (27)	13⋅5 (73)	9⋅2 (12)	
Fast food consumption				
Decreased	21⋅6 (32)	38⋅3 (207)	35⋅9 (47)	**0⋅004**
Increased	13⋅5 (20)	13⋅1 (71)	13⋅0 (17)	
Homemade pastries				
Decreased	14⋅9 (22)	13⋅3 (72)	14⋅5 (19)	**0⋅042**
Increased	23⋅6 (35)	32⋅2 (174)	40⋅5 (539	
Industrial bakery products				
Decreased	19⋅6 (29)	28⋅1 (152)	26⋅7 (35)	0⋅083
Increased	12⋅8 (19)	17⋅4 (94)	17⋅6 (23)	
Sweets				
Decreased	9⋅5 (14)	18⋅5 (100)	14⋅5 (19)	**0⋅017**
Increased	28⋅4 (42)	33⋅5 (181)	35⋅9 (47)	
Cake				
Decreased	16⋅2 (24)	21⋅4 (116)	19⋅1 (25)	0⋅111
Increased	17⋅6 (26)	23⋅3 (126)	27⋅5 (36)	
Sugar/Toffees				
Decreased	14⋅9 (22)	19⋅4 (105)	16⋅8 (22)	0⋅094
Increased	10⋅1 (15)	17⋅4 (94)	17⋅6 (23)	
Snacks				
Decreased	15⋅5 (23)	17⋅6 (95)	23⋅7 (31)	0⋅192
Increased	13⋅5 (20)	16⋅5 (89)	23⋅7 (31)	
Sweetened Cereals/Cereal Bars				
Decreased	9⋅5 (14)	15⋅9 (86)	18⋅3 (24)	**0⋅023**
Increased	13⋅5 (20)	15⋅0 (81)	22⋅1 (29)	
Honey				
Decreased	9⋅5 (14)	12⋅9 (70)	12⋅2 (16)	0⋅373
Increased	20⋅9 (31)	18⋅5 (100)	25⋅2 (33)	
Pekmez				
Decreased	11⋅5 (17)	10⋅9 (59)	13⋅0 (17)	0⋅931
Increased	16⋅2 (24)	15⋅7 (85)	17⋅6 (23)	

Data are presented as the number of persons (%).

†† The foods which were given one score when their consumption decreased based on the MEDAS scoring.

The bold indicates statistically significant values (*P* < 0⋅05).

Additionally, the changes in the consumption of foods differed according to gender. Women's consumption of dry fruits, fresh vegetables, pasta and grains, whole grain foods, milk and yogurt, cheese, fish, legumes, white meat, homemade food and homemade pastries were increased more than men's (*P* < 0⋅05) (Supplementary Figure S1). Moreover, women's fast food and take-away food consumption decreased more than men's (*P* < 0⋅05) (Supplementary Figure S2).

The changes in drink consumption according to FCV-19S classification are given in Table 5. Water consumption increased by nearly 45 % of the respondents in all groups. Moreover, approximately 35 % of the respondents with high FCV-19S increased their black tea, herb tea and coffee consumption. On the other hand, approximately 10 % of the respondents with high FCV-19S stated decreased consumption of wine, beer and other alcoholic beverages, while about 15 % of the respondents with low FCV-19S decreased the consumption of these alcoholic beverages. Furthermore, women's consumption of black tea, herbal tea and coffee increased more than men's during the pandemic (*P* < 0⋅05) (Supplementary Figure S3).

**Table 5.** Changes in beverage consumption according to FCV-19S during the pandemic

	Level of FCV-19S			
	Low fear (*n* 148)	Moderate fear (*n* 541)	High fear (*n* 131)	*P*-value
Water				
Decreased	5⋅4 (8)	8⋅7 (47)	10⋅7 (14)	0⋅518
Increased	43⋅9 (65)	45⋅7 (247)	44⋅3 (58)	
Black Tea				
Decreased	6⋅1 (9)	9⋅6 (52)	10⋅7 (14)	0⋅209
Increased	31⋅8 (47)	38⋅6 (209)	35⋅9 (47)	
Herb Tea				
Decreased	11⋅5 (17)	10⋅9 (59)	7⋅6 (10)	0⋅258
Increased	21⋅6 (32)	29⋅8 (161)	30⋅5 (409	
Coffee				
Decreased	15⋅5 (23)	16⋅8 (91)	11⋅5 (15)	0⋅229
Increased	26⋅4 (39)	29⋅8 (161)	37⋅4 (49)	
Sugary/Sparkling Beverages				
Decreased	16⋅2 (24)	22⋅7 (123)	17⋅6 (23)	0⋅094
Increased	10⋅8 (16)	12⋅4 (67)	18⋅3 (24)	
Wine/Beer				
Decreased	14⋅9 (22)	12⋅9 (70)	13⋅0 (17)	0⋅716
Increased	5⋅4 (8)	5⋅9 (32)	3⋅1 (4)	
Other Alcoholic Beverages				
Decreased	16⋅9 (25)	12⋅8 (69)	12⋅2 (16)	0⋅571
Increased	4⋅7 (7)	5⋅9 (32)	3⋅8 (5)	

Data are presented as the number of persons (%).

## Discussion

The present study investigated the possible relationships between the fear of COVID-19, adherence to MD and dietary changes during the third wave of COVID-19 pandemic in Turkey. MD is among the healthiest dietary patterns as it includes a variety of healthy foods such as vegetables, legumes and whole grains and has reducing effects on cardiovascular disease and overall mortality^(23)^. Studies have shown that greater adherence to MD was linked with numerous positive effects on health^(24)^; within this respect, having a diet compatible with MD during the pandemic might be beneficial^(25)^. On the other hand, stress, fear and anxiety experienced by individuals during the pandemic might negatively affect healthy food choices and adherence to the MD. In addition, restrictions such as lockdowns during the pandemic period might affect nutritional behaviours^(26,27)^. In the present study, most of the participants adhered to MD moderately; however, an earlier study on Turkish adults during pandemic displayed that most participants were non-adherent to MD^(28)^. Previous studies conducted on different populations demonstrated that pandemic led people to adopt healthier dietary habits and greater adherence to MD^(16,29,30)^.

In the present study, contrary to the high MD adherence of the participants with high fear, worsened lifestyle, increased appetite, total food consumption and an increased number of meals were more common according to their statements. Notably, while most of the participants stated taking supplements daily, a significant portion of those who took supplements stated that they started taking supplements during the pandemic. Previous studies examining the changes in lifestyles and food consumption of adults during the pandemic have shown that they were affected differently by the pandemic depending on the pandemic's period and the participants’ characteristics^(31–33)^. Similar to the effects of fear, one of the psychological factors we examined in our study, researchers pointed out that psychological changes, stress, anxiety, depression and beliefs related to health and nutrition were linked with altered eating habits and lifestyle during pandemic^(34,35)^.

Meller *et al.* showed that fear of COVID-19 had a linear relationship with health outcomes^(36)^. Moreover, researchers found that higher fear of COVID-19 was common among women^(36–39)^. According to Broche-Pérez *et al.*, gender substantially predicted the degree of dread of COVID-19 and women were more psychologically vulnerable during the pandemic^(40)^. Female gender is thought to be significantly associated with a greater psychological impact of the pandemic and higher levels of stress, anxiety, depression and perceived stress^(41)^. Notably, women had significantly higher fear of COVID-19 than men in the present study. In addition, significant differences related to eating behaviour were also observed between the genders in this study. Women seem to tend to consume healthier foods than men. These findings align with previous studies reporting higher adaptation of women to healthier eating behaviours during the pandemic^(42,43)^. Moreover, women had higher MD adherence than men in this study, in line with previous studies related to pandemic and MD^(18,43)^. This could be explained by the fact that those with high fear levels were motivated to consume a healthier diet to improve their health. On the other hand, although our results showed that participants with higher fear of COVID-19 had higher adherence to MD, the changes in the consumption of foods among the MD components did not generally differ except for increasing sweetened cereals/bars, pasta and homemade bread/pastries and decreasing take-away food and fast food according to the level of fear. These changes were partially consistent with the literature, which reports conflicting outcomes. Concordantly, it was thought that the chance of cooking at home during the pandemic period and the decrease in ready-to-eat food consumption were also effective in increasing adherence to MD^(29)^.

The present study has some strengths and limitations that should be addressed. The study's cross-sectional design led us to examine only the relationship between fear of COVID-19, adherence to MD and eating habits, whereas causation could not be assessed. The online self-reported survey limited the degree of representativeness of the participants. However, online surveys are considered suitable methods to collect data during the pandemic. The study's online design also limited the older population's participation. Moreover, reaching a larger size of the sample could have increased the representativeness of the respondents. Due to the lack of participants’ pre-pandemic data, the pandemic's effect was assessed based on the participants’ statements. On the other hand, inclusion of a considerably large number of participants living in different areas of Turkey and using validated scales are among the strengths of the study.

In conclusion, the present study's findings demonstrated that Turkish adults had moderate adherence to MD during the third wave of the COVID-19 pandemic. Participants’ food consumption and eating habits differed according to the fear of COVID-19 levels. The fear seems that trigger the consumption of not only sweetened foods but also homemade foods. Furthermore, women had higher levels of fear than men. In addition, during the pandemic, women were more likely to adopt a healthier diet than men, with increased consumption of homemade foods, white meats, legumes, fish, cheese, milk and yogurt, whole grain food, pasta and grains, fresh vegetables, and dry fruits and decreased consumption of fast food and take-away foods. Although causality cannot be assumed in this study, the present results may help develop nutritional recommendations to prevent the adverse effects of future outbreaks on nutrition and health.

## Supplementary material

For supplementary material accompanying this paper visit https://doi.org/10.1017/jns.2023.40. click here to view supplementary material
