# Supplementary material for: Adherence to Mediterranean diet and dietary changes according to the fear of COVID-19 during the pandemic: a cross-sectional study
Source: J Nutr Sci. 2023 May 8;12:e56. doi: 10.1017/jns.2023.40 (PMC10173089; doi:10.1017/jns.2023.40)
Supplement: Supplementary file 1 [file S204867902300040Xsup001.docx]

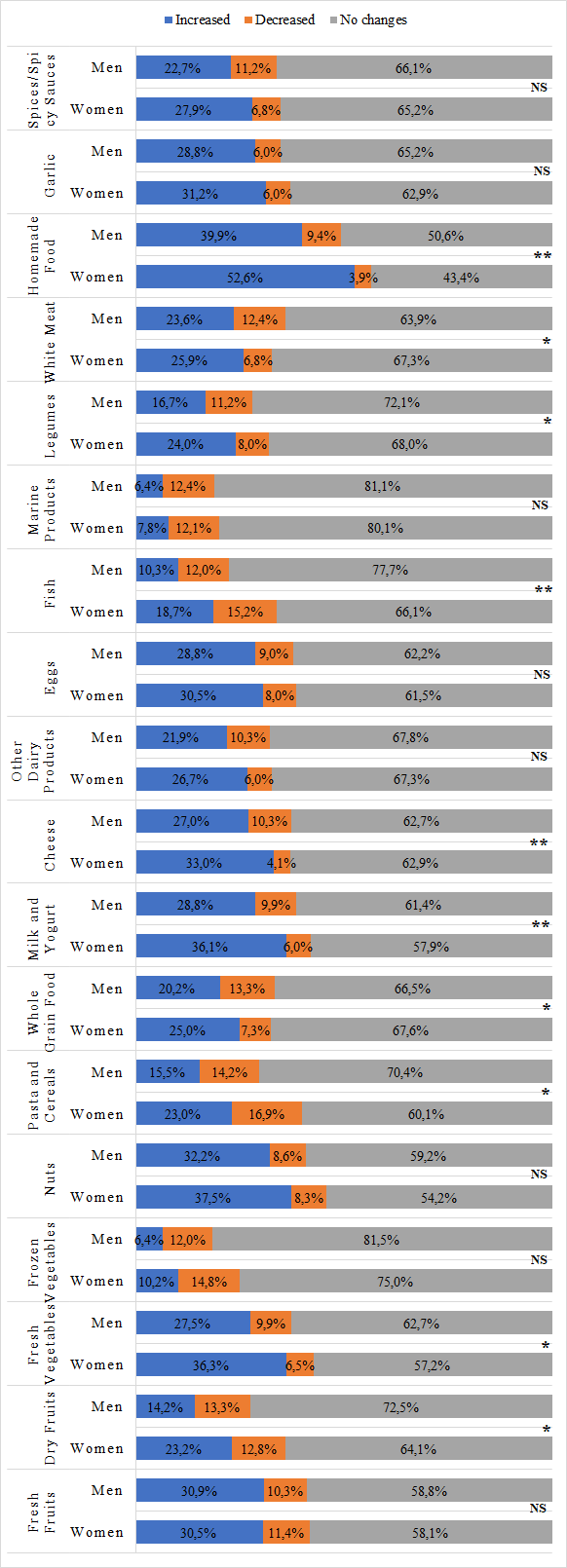


**Supplementary Figure 1.** The percentage of change in positive MEDAS scored-foods consumption by gender during COVID-19. *p<0.05, **p<0.01, NS; not significant (p>0.05).


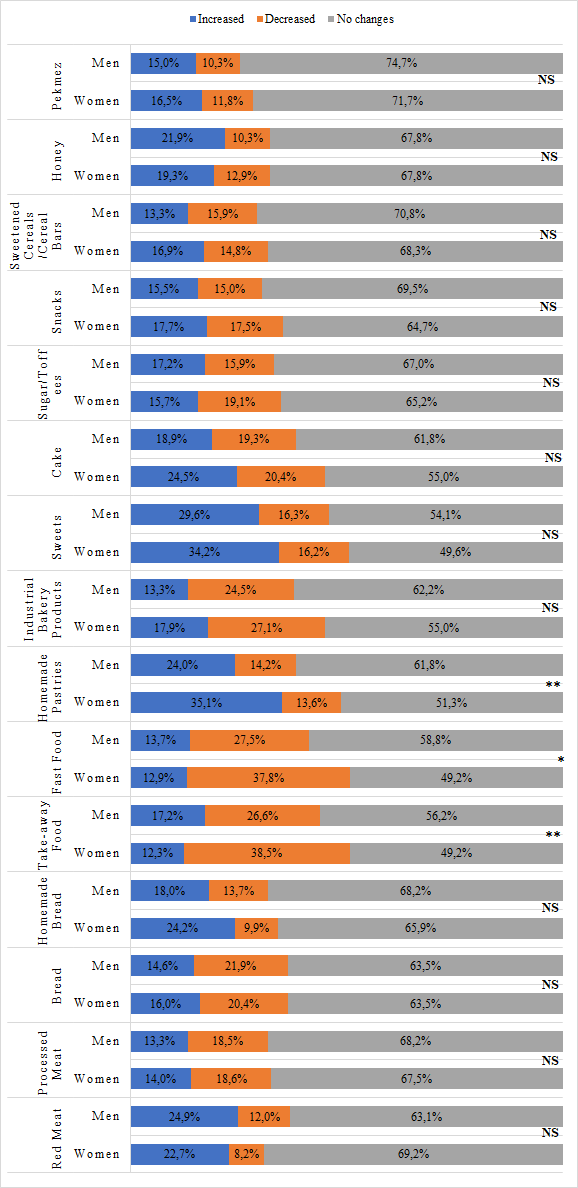


**Supplementary Figure 2.** The percentage of change in other foods consumption by gender during COVID-19. *p<0.05, **p<0.01, NS; not significant (p>0.05).


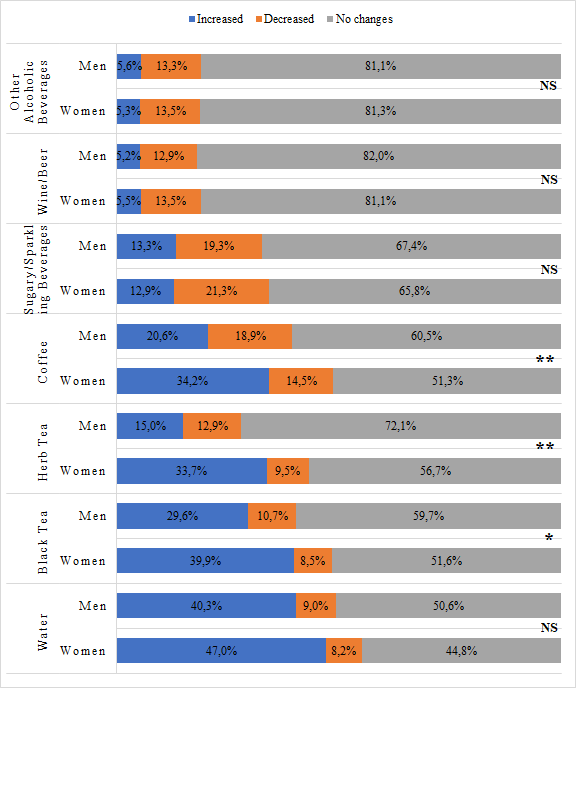


**Supplementary Figure 3.** The percentage of change in beverage consumption by gender during COVID-19. *p<0.05, **p<0.01, NS; not significant (p>0.05).
